# Magnetic Properties of Nitrogen-Doped Graphene Induced by Dopant Configurations

**DOI:** 10.3390/nano15221694

**Published:** 2025-11-09

**Authors:** Madhuparna Chakraborty, Gregory Jensen, David C. Ingram, Eric Stinaff, Wojciech M. Jadwisienczak

**Affiliations:** 1 School of Electrical Engineering and Computer Science, Ohio University, Athens, OH 45701, USA; 2 Department of Physics and Astronomy, Ohio University, Athens, OH 45701, USA

**Keywords:** graphene, non-metallic magnetism, nitrogen doping, annealing, nanomaterial

## Abstract

In this study, we experimentally demonstrate that the magnetic properties of nitrogen-doped graphene (NG) are influenced by the configuration of nitrogen dopants, namely graphitic, pyridinic, and pyrrolic, along with the overall nitrogen concentration. The NG materials were prepared via a two-step thermal treatment process. The first step involved heating in ammonia at 400 °C, followed by a second post-annealing step at 600 °C. Scanning Electron Microscopy–Energy Dispersive X-ray Spectroscopy (SEM–EDS) analysis performed at 25 μm resolution confirmed uniform elemental distribution across the samples. X-ray photoelectron spectroscopy (XPS) revealed that while the total nitrogen content decreased from 11.9 at.% in NG to 5.5 at.% in the post-annealed sample, the ratio of graphitic to pyrrolic nitrogen increased from 0.4% to 3.8% and the ratio of graphitic to pyridinic nitrogen increased from 0.8% to 2.5%. Raman spectroscopy confirmed the presence of prominent D and G bands at ~1352 cm^−1^ and ~1589 cm^−1^, respectively, along with a 2D band at ~2692 cm^−1^, indicating the presence of few-layered graphene and defect-related features. The $\frac{I_{D}}{I_{G}}$ ratio increased from 1.12 to 1.27 in the post-annealed sample, indicating increased disorder after annealing. Magnetic characterization showed a marked enhancement in the magnetic properties with increased graphitic nitrogen content. The saturation magnetization (*M_s_*) reached 0.13 emu g^−1^, ~42% higher than that of the material heated in ammonia, with the coercivity increasing from 40 Oe to 750 Oe. These results emphasize the pivotal role of nitrogen configuration in the graphene host, specifically the promotion of graphitic nitrogen species, in tailoring the ferromagnetic response of NG.

## 1. Introduction

**Figure 1:**
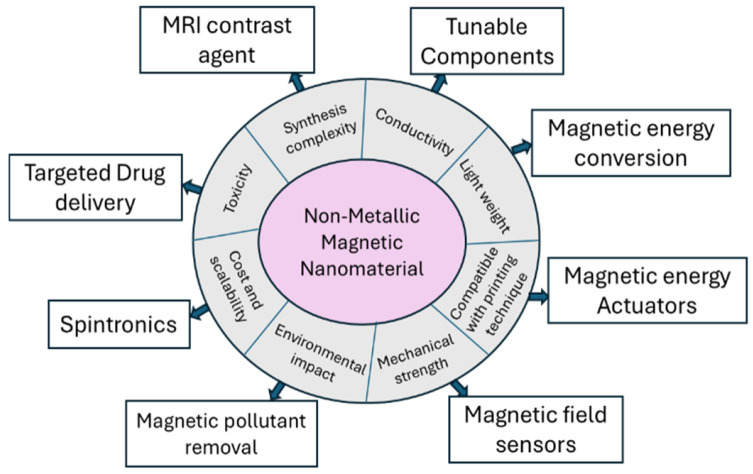
A schematic overview illustrates the advantages and diverse applications of nonmetallic magnetic nanomaterials.

Magnetism in non-metallic materials represents an emerging field in material science, challenging the traditional view that magnetism is predominantly associated with transition metals and rare-earth elements [1]. In recent years, carbon-based systems such as nitrogen-doped graphene (NG) have demonstrated intrinsic magnetic behavior arising from localized spins, defects, or heteroatom-induced electronic asymmetry [2,3,4,5,6,7,8]. Unlike metallic magnets, which rely on unpaired *d*- or *f*-electrons, nonmetallic magnets often derive their magnetic moments from spin delocalization [2], creating opportunities for developing next-generation spintronic devices, biocompatible magnetic materials, and components for quantum technologies using sustainable and abundant nonmetallic resources. Thus, one may anticipate that non-metallic magnetic materials offer numerous advantages and diverse applications across emerging technologies, some of which are shown in Figure 1.

Many non-metallic systems, particularly those based on carbon nanostructures like graphene, can exhibit high electrical conductivity in addition to magnetic properties. This rare combination enables multifunctional applications, allowing the development of tunable and reconfigurable components such as inductors, antennas, and filters whose behavior can be dynamically controlled via external magnetic fields, making them ideal for building circuits and in spintronics devices [9,10]. Unlike metals such as iron, nonmetals do not undergo oxidation over time, thereby preserving their performance. The nonmetallic materials’ inherent low density makes them exceptionally lightweight, a major advantage for wearable electronics, aerospace, satellite, and biomedical devices where weight is critical [11]. Embedding magnetic particles enables magnetic energy conversion, harvesting energy from motion or ambient vibrations, and can also be used for electromagnetic wave absorption [12,13]. Mechanically, these materials also offer superior flexibility and durability under strain, which is essential for applications such as soft robotics, stretchable circuits, and medical implants. In wearable systems, it allows real-time health monitoring, motion tracking, and posture sensing [14,15]. Magnetic energy conversion uses magnetic materials to turn mechanical motion into electrical energy, especially in systems that need to be bendable and stretchable. This is often done by mixing magnetic particles into soft polymer materials, so when the material moves or bends it generates electricity through magnetic induction [16]. Non-metallic magnetic nanomaterials are suitable for making inks for flexible electronics as they can be easily dispersed in common ink solvents, making them well-suited for printing methods like inkjet, aerosol jet, and screen printing [17]. This makes it possible to produce flexible electronic components of scale using roll-to-roll manufacturing, making them cost-effective. Environmentally, non-metal magnetic materials reduce reliance on transition metals and rare-earth elements, often being synthesized from abundant, low-toxicity precursors via green processes. They are reusable in water purification systems, where magnetic separation allows selective removal of heavy metals and organic contaminants [18]. Their biocompatibility and low cytotoxicity further support safe integration into healthcare devices. In targeted drug delivery, magnetic fields can guide these materials to specific sites in the body, while in hyperthermia therapy, they enable localized heating for cancer treatment [19]. These materials are also scalable and cost-effective, compatible with easy processing. Furthermore, unlike traditional metallic magnets, nonmetallic magnetic materials can often be synthesized using simpler, low-temperature solution processes, avoiding the need for high-vacuum or high-energy metallurgical methods. In imaging, tunable magnetic nanoparticles enhance MRI contrast with lower toxicity. For storage, they enable dense, efficient data retention by controlling magnetic domains [20]. For quantum computing, they help manipulate spin-based qubits with precision [21], and in spintronics, they improve energy efficiency and switching speed [22]. Additionally, magnetic fields can enhance catalytic performance in processes like water splitting and CO_2_ reduction, supporting sustainable energy technologies [23].

Graphene, with a single layer of *sp^2^*-bonded carbon atoms, exhibits remarkable electronic and mechanical properties [24]. However, its inherent diamagnetism limits direct magnetic applications. Nitrogen doping has emerged as an effective strategy to introduce magnetism by altering the electronic structure and creating spin polarization [2,3,5]. Among the various nitrogen bonding configurations, the influence of graphitic-, pyridinic-, and pyrrolic-N on the magnetic properties of graphene remains a topic of active investigation [2,3,5,25,26,27,28,29,30,31]. Among the different N-configurations, pyridinic-N is located at the edges or defects of the lattice, where it bonds with two carbon atoms in an *sp*^2^ configuration and contributes one electron to the *π*-system [32]. Pyrrolic-N, in contrast, is found in five-membered rings, where it bonds with two carbons forming a *sp*^3^ configuration. In this case, the nitrogen contributes two electrons to the *π*-system through its delocalized lone pair, thereby enhancing the carrier density and electronic conjugation [32]. Graphitic-N, also referred to as quaternary-N, substitutes for a carbon atom within the basal plane and bonds to three neighboring carbons in an *sp*^2^ configuration [32]. It contributes one electron to the *π*-system while seamlessly integrating into the lattice, thereby modulating the charge distribution. Motivated by the key role of nitrogen’s electronic configuration in determining magnetic properties, we focused on the specific bonding configurations of nitrogen that govern the magnetic behavior of NG, rather than on overall nitrogen concentration alone. Prior experimental studies have indicated that nitrogen doping can generate magnetic properties in graphene [3,25,26,27,28,29,30,31]. Li et al. [25], Miao et al. [26], Qin et al. [27] attributed this enhancement primarily to the pyrrolic-N bonding configuration. In contrast, Błoński et al. [3], Fu et al. [5] have argued that graphitic-N is the dominant contributor to the ferromagnetic state, while pyridinic-N, pyrrolic-N play only a minor role. Further, Ito et al. [33] reported that nitrogen incorporation can reduce the magnetic response when pyrrolic-type sites are formed. These differing conclusions show the ambiguity regarding the role of individual nitrogen bonding configurations in governing magnetism in the NG.

Doping graphene with nitrogen is challenging, and various approaches were reported in the past. Wang et al. [34] reported that nitrogen doping to a perfect graphene sheet requires an activation energy barrier of 107.2 kcal mol^−1^, making direct substitution difficult even at high temperatures. However, chemical schemes like defluorination can drastically lower this barrier and enable nitrogen incorporation, motivating the use of fluorinated graphite (FG) as an agent for nitrogen doping. Under an atmosphere of protonic nucleophiles such as NH_3_ at high temperature, C–C bond dissociation becomes feasible through defluorination, opening pathways for doping [35]. Li et al. [36] described the mechanism of nitrogen doping in FG as happening through three steps. The first step includes amino functionalization, where NH_3_ adsorption (−5.1 to −5.2 kcal mol^−1^) leads to hydrogen transfer to a neighboring fluorine, yielding a C–NH_2_ group and HF release. This proceeds through a low barrier of 4.4 kcal mol^−1^, making amino grafting highly favorable. The second step involves further defluorination, which drives intramolecular cyclization of the amino group into ethylenimine intermediates. This reaction is strongly exothermic (−18.7 to −14.3 kcal mol^−1^) with barriers of 14.0–21.7 kcal mol^−1^, respectively. The final step is the doping stage, which involves embedding ethylenimine moieties into the graphene lattice, forming pyridinic, pyrrolic, or graphitic motifs depending on the local defects. Multi-vacancy defects favor pyridinic- and pyrrolic-N, while isolated single vacancies stabilize graphitic-N. This defect-governed selectivity could be a driving mechanism for controlling nitrogen configurations in graphene.

In this work, we investigated the NG achieved from FG in a two-step thermal conversion process to examine how nitrogen bonding configurations influence the magnetic behavior of graphene. Specifically, X-ray photoelectron spectroscopy (XPS) was employed to identify and quantify the distinct nitrogen bonding configurations introduced during the conversion of FG to NG. Vibrating sample magnetometry (VSM) measurements revealed how the dopant configurations directly influence the magnetic response. This approach establishes a clear structure–property relationship, demonstrating that the emergence of intrinsic ferromagnetism in graphene-based systems can be rationally tailored through dopant engineering. Our findings provide critical insight into how graphitic, pyridinic, and pyrrolic nitrogen species contribute differently to the stabilization of magnetic ordering. While previous studies have primarily emphasized total nitrogen content, the fundamental role of specific nitrogen bonding motifs in governing magnetic behavior has remained largely unexplored experimentally. This work deepens understanding of how NG atomic-level doping chemistry influences magnetic functionality, paving the way for the design of sustainable, metal-free magnetic materials for next-generation applications.

## 2. Materials and Methods

### 2.1. Synthesis of Nitrogen-Doped Graphene

Here, NG samples were synthesized via a two-step process involving thermal treatment of FG under an ammonia atmosphere, followed by annealing under inert conditions. Commercial FG powder from Sigma-Aldrich (St. Louis, MO, USA), CAS No. 51311-17-2 was used as the starting material. In a typical setup, high-purity argon (Ar, 60 sccm) is used for purging to eliminate residual air. The furnace temperature was then increased to the desired reaction temperature (400–600 °C). Once the target temperature was reached, the sample was rapidly introduced into the hot zone under a continuous flow of high-purity ammonia gas (NH_3_, 99.999%, 180 sccm). The reaction was maintained for 1 h to facilitate nitrogen incorporation into the graphene lattice. After completion, the sample was removed from the hot zone and allowed to cool to room temperature. Samples were designated based on the synthesis temperature, NG-400 (400 °C), NG-500 (500 °C), and NG-600 (600 °C), respectively. Hereafter, e.g., NG-400 implies S1. To further modulate the nitrogen bonding configuration, the NG-400 sample underwent a secondary annealing step. The as-prepared NG material was annealed at 600 °C for 1 h under an Ar atmosphere (60 sccm) and is labeled as S2 sample. We focus on the annealing of NG-400, as higher-temperature annealing of NG-500 and NG-600 led to a deterioration in magnetic saturation, which is the desired characteristic for optimization, as shown in Figure S1 and Table S1 in the Supplementary Information (SI).

### 2.2. Investigation Methods

Surface morphology and elemental composition of the samples were analyzed using a TESCAN VEGA scanning electron microscope (SEM) integrated with an Oxford Instruments energy-dispersive X-ray spectroscopy (EDS) system. The measurements were carried out at an accelerating voltage of 15 kV under high-vacuum conditions. Elemental mapping and quantitative EDS analysis were performed using the AZtec software suite (5.1, Oxford Instruments, Abingdon, UK), which enabled accurate identification and spatial distribution of constituent elements across the sample surface. X-ray Photoelectron Spectroscopy (XPS) was performed using a Kratos XSAM 800 electron spectrometer (1484 Kα Al X-ray source, 120 W power, pass energy 40 eV, NY, USA) to investigate the chemical states of elements in NG. The C1s peak at 284.8 eV was used for calibration, and data were processed in CasaXPS. The structural properties of samples were investigated by Raman spectroscopy using a Senterra-I from Bruker Corporation (Karlsruhe, Germany) equipped with a confocal microscope with a magnification of ×100, a laser source operating at 532 nm, and a 400 groove/mm grating, resulting in a spectral resolution of 3 cm^−1^. Low power density of 0.52 MWcm^−2^ of laser source was used so as not to heat the samples in situ under the laser beam. Magnetic properties were analyzed using a Physical Property Measurement System (PPMS) DynaCool system with a Vibrating Sample Magnetometer (VSM) from Quantum Design Inc. (San Diego, CA, USA). The magnetic materials were studied using a powder capsule with background correction applied. The samples were measured in the range of 2 K–350 K.

## 3. Results and Discussion

### 3.1. Elemental Analysis Using SEM/EDS

**Figure 2:**
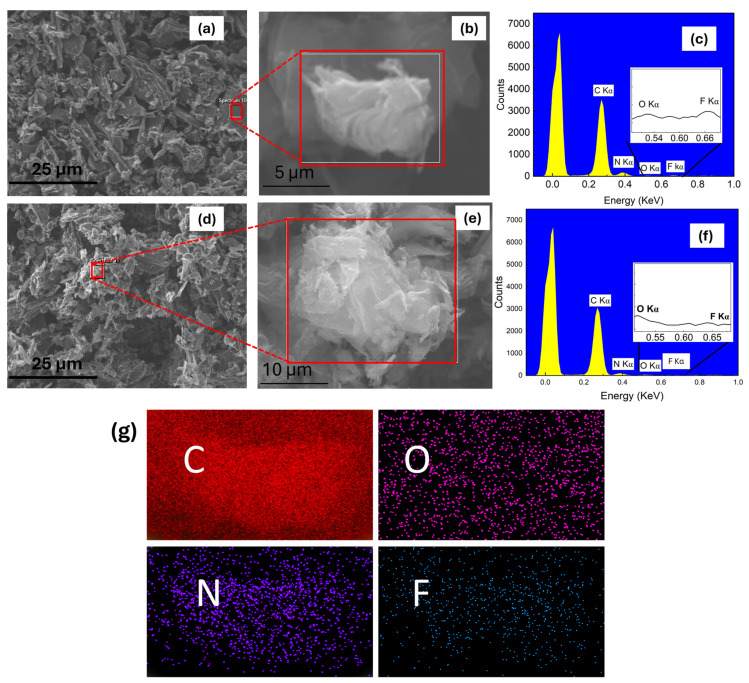
Scanning electron microscopy (SEM) micrographs and energy-dispersive X-ray (EDX) spectra of S1 and annealed S2 samples. (**a**) Low-magnification SEM image of S1 sample showing sheet-like morphology. (**b**) Zoomed-in image of (**a**). (**c**) EDS spectrum corresponding to the boxed region in (**a**), confirming the presence of C, N, O, and F in the S1 sample. The insert in (**c**) shows a magnified view of the region containing residue F content. (**d**) SEM image of S2 sample illustrating retention of wrinkled morphology. (**e**) Zoomed-in image of (**d**). (**f**) EDX spectrum from the boxed region in (**d**) and insert showing the magnified portion of F content (**g**). Elemental mapping of C, N, O, and F distributions for the post-annealed S2 sample region is shown in (**e**).

To investigate the impact of nitrogen doping and subsequent thermal treatment on the structural and elemental characteristics of fluorinated and nitridated graphene samples, we performed SEM and EDS analyses on samples S1 and S2. The corresponding results are shown in Figure 2 and Table 1. In Table 1, the k-ratio refers to the ratio of characteristic X-ray intensities. As illustrated in Figure 2a, the S1 sample exhibits an irregular flake-like morphology with large lateral dimensions and a rough surface.

SEM images of the S1 sample display roughness across the surface, which can be attributed to local changes in electron distribution around dopant sites. This occurs because nitrogen (3.04) is more electronegative than carbon (2.55) and has a different atomic radius, leading to charge imbalance, bond length variations, and strain within the graphene sheet, all of which make the rough features as observed in the SEM image [37,38]. Figure 2b shows a zoomed image of Figure 2a depicting thin and crumpled layers, randomly aggregated. The EDS spectrum of S1 sample shown in Figure 2c confirms the presence of C, N, and O, along with trace amounts of F carried over from the precursor. The observed peaks are consistent with standard reference values [37,38,39,40]. Figure 2d shows the morphology of sample S2. After thermal treatment, the material maintains its flake-like rough structure of the doped graphene framework. Although the surface remains non-uniform, subtle changes in the flakes suggest partial modification of the morphology of the sample. The S2 sample seems to have a higher surface-to-volume ratio, with a noticeable reduction in thickness. The increased surface area indicates a greater degree of graphite exfoliation in the S2 sample. Figure 2e shows the zoomed image of Figure 2d, which shows the presence of multiple layers. The EDS spectrum of the S1 sample in Figure 2f confirms the presence of C, N, and O, while F is slightly detectable, as also shown in Table 1, suggesting its major removal. The elemental mapping in Figure 2g shows a uniform distribution of nitrogen, indicating successful and homogeneous doping throughout the graphene matrix. SEM and EDS analyses reveal that the samples exhibit a rough, irregular surface morphology, likely arising from nitrogen incorporation, which perturbs the graphene lattice. Elemental mapping confirms that nitrogen is uniformly distributed, indicating that doping likely creates structural variations. Subsequent annealing retained the overall morphology and elemental homogeneity of the sample.

### 3.2. XPS Characterization of Elemental and Chemical States

**Figure 3:**
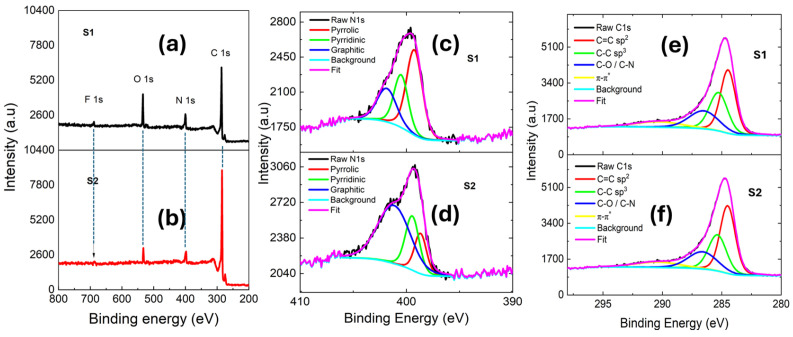
XPS wide-scan spectra for (**a**) S1 and (**b**) S2 samples. Peaks corresponding to core levels of C1s, N1s, O1s, and F1s are labeled. The S2 sample shows a sharper C1s peak and a significantly reduced N1s intensity, indicating lower total nitrogen content. (**c**) High-resolution spectra of N1s for the S1 sample with dominant pyrrolic-N and pyridinic-N contents. (**d**) High-resolution spectra of N1s for the S2 sample showing a dominant graphitic-N proportion. (**e**) High-resolution spectra of C1s for the S1 sample. (**f**) High-resolution C1s spectra for the S2 sample.

XPS spectra of the S1 and S2 show characteristic peaks corresponding to C1s, N1s, O1s, and F1s, as shown in Figure 3a,b. Deconvolution of the N1s peak shown in Figure 3c,d reveals the presence of three distinct nitrogen species: pyrrolic-N, pyridinic-N, and graphitic-N, respectively. High-resolution XPS spectra were deconvoluted using a Shirley background and fitted with Voigt-based (LA) line shapes, with all elemental regions treated consistently using the Shirley background subtraction and Gaussian–Lorentzian fitting [3,5,8]. The residual standard deviation (RSD) is approximately 0.98. Table 1 and Table 2 show comparable elemental levels, with the main variation observed in oxygen. The detected oxygen signals in XPS may arise from surface contamination during ex situ measurement, which could account for this difference.

The XPS survey spectra of the S1 and S2 samples are shown in Figure 3a,b. As shown in Table 2 [3,5,8], the C/N ratio increases from 4.8 at. % to 5.9 at.% as the synthesis temperature increases. The nitrogen concentration increased with synthesis temperature, indicating enhanced reactivity and greater incorporation of nitrogen into the graphene lattice. With increasing synthesis temperature, more defluorination occurs as seen in Table 2, facilitating the incorporation of amino groups into the graphene lattice. The concentration of pyrrolic-N is highest at lower synthesis temperatures, NG-400 (S1), but decreases progressively with increasing temperature as seen in NG-500 and NG-600. In contrast, the pyridinic-N content increases with synthesis temperature, accompanied by a slight increase in the graphitic-N contribution. Guo et al. [41] have shown that the distribution of nitrogen configurations was found to be strongly temperature dependent, evolving systematically with increasing pyrolysis temperature. Pyrrolic-N exhibits poor thermal stability and readily converts into pyridinic-N and graphitic-N species. This transformation is thermodynamically driven, as the formation energy of pyrrolic-N configuration (3.28 eV) is substantially higher than that of pyridinic-N (1.15 eV) and graphitic-N (0.98 eV) [41]. Graphitic-N represents the most stable configuration. Upon annealing the sample, the overall nitrogen content decreases, likely due to C–N bond breakage and subsequent nitrogen loss. As shown in Table 2, the S2 sample exhibits a dominant graphitic-N contribution of 60.6%, accompanied by a reduction in pyrrolic-N and pyridinic-N fractions. Pyrrolic-N and pyridinic-N species are typically located at edge sites, whereas graphitic-N is formed within the basal plane, as mentioned earlier [32]. At elevated temperatures, pyrrolic-N and pyridinic-N configurations tend to lose nitrogen, with a fraction converting into a more stable graphitic-N configuration. Annealing at 600 °C (S2 sample) led to an increased fraction of graphitic-N, whereas direct synthesis at 600 °C (NG-600) did not yield a comparable enhancement. This suggests that post-annealing promotes structural rearrangement and stabilization of graphitic sites, rather than their direct formation during the synthesis process. The synthesis process likely generated multi-vacancy defects that promoted the formation of pyrrolic-N and pyridinic-N, while subsequent annealing favored the stabilization of the more thermodynamically stable graphitic-N.

The deconvoluted C1s spectra in Figure 3e,f display peaks at 284.5, 285.1, 286.5, and 290.3 eV, corresponding to C=C, C-C, C–N/C–O, and π-π* bonds, respectively [42]. The C=C *sp*^2^ component, associated with pyridinic-N and graphitic-N configurations, increased from 15.3% to 25.1% in sample S2, while the *sp*^3^ C-C component, corresponding to pyrrolic-N, decreased from 18.6% to 13.6% post annealing. The C1s spectrum of the sample demonstrates most of the carbon C–C bonds as a graphene structure. The ratios of the different nitrogen dopants remain relatively stable for samples synthesized at different synthesis temperatures, which is attributed to the loss of certain amino groups in the precursor. However, the ratio of the different nitrogen dopant configurations exhibits a different trend after the second annealing step. The N1s spectra grown at 500 °C and 600 °C are shown in Figure S2 in the SI. For those samples, the pyrrolic-N and pyridinic-N configurations are seen as the dominant nitrogen dopant types.

### 3.3. Raman Analysis of Structural Properties

**Figure 4:**
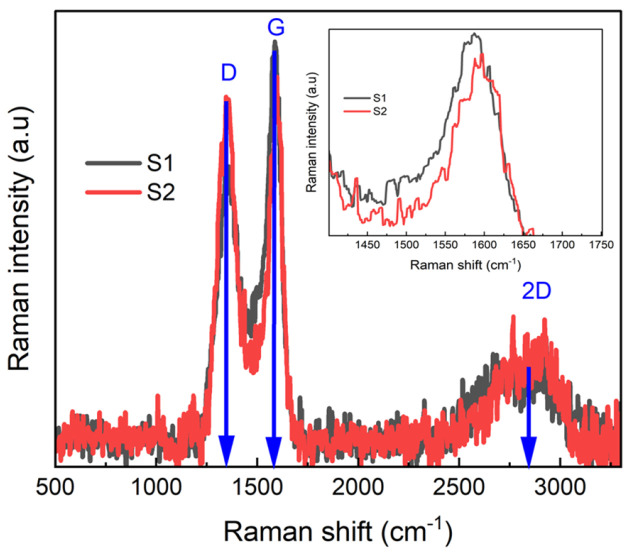
Raman spectra of S1 and S2 samples showing the characteristics of D and G bands along with the 2D band.

Figure 4 shows the Raman spectra of S1 and S2 samples. Both samples exhibit distinct D and G bands at ~1352 cm^−1^ and ~1589 cm^−1^, respectively. The D band originates from structural defects such as edges, vacancies, bonding disorders, and heteroatom incorporation, primarily due to nitrogen doping for our sample, whereas the G band corresponds to the *E*_2_*_g_* phonon mode of *sp*^2^-bonded carbon [43]. A pronounced 2D band indicates the formation of few-layer graphene, while the broad peak results from the overlap between the 2D ∼2692 cm^−1^ and D + D′ ∼2936 cm^−1^ bands [44]. The deconvoluted peaks are presented in Figure S3 of the SI. Compared to pristine graphene (D: ~1350 cm^−1^, G: ~1580 cm^−1^, 2D: ~2690 cm^−1^) [45], both S1 and S2 samples show an upshift in the D, G, and 2D bands, which can be attributed to Fermi-level modification induced by nitrogen dopants [46,47].

According to Solati et al. [48], Raman peak positions are sensitive to both the amount and configuration of nitrogen dopants. The G peak is sensitive to stress within C–C bonds. In the case of the S2 sample with lower nitrogen concentration, graphitic-N causes negligible bond length changes, whereas pyridinic-N and pyrrolic-N configurations disrupt lattice symmetry by replacing carbon atoms and reducing the coordination of neighboring carbon atoms, leading to bond modifications, respectively. The increased graphitic-N concentration in the S2 sample, as observed in XPS, likely contributes to the G band shift with respect to the S1 sample, as seen Figure 4 inset. Overall, the upshift and broadening of these bands, especially the D band, are consistent with structural distortions caused by heteroatom substitution, such as C–N bond formation [47,49]. The full-width half-maxima (FWHM) Γ values of the bands for D, G, and 2D are 108.4 ± 0.7, 53.8 ± 0.5, and 192.0 ± 1.5 cm^−1^, respectively, for sample S1. The intensity ratio of the D to G band $\frac{I_{D}}{I_{G}}$ [43] of S1 is 1.12 ± 0.01. The annealed graphene sample S2 shows narrowing of all bands, with the *Γ* values for the D, G, and 2D bands being 82.2 ± 0.8, 37.3 ± 0.4, and 187.7 ± 2.5 cm^−1^, respectively. There is an increase in $\frac{I_{D}}{I_{G}}$ ratio to 1.27 ± 0.09 observed in sample S2. We notice that the S2 sample, with low nitrogen doping levels compared to the S1 sample, shows reduced Γ widths of the G band. The intensity ratio $\frac{I_{D}}{I_{G}}$ is commonly used to estimate defect density in graphene-based materials [43,44,47]. Although the overall nitrogen at.% decreases after annealing, as indicated by XPS, the slight increase in $\frac{I_{D}}{I_{G}}$ implies possible rearrangement after thermal treatment.

In the case of a sample containing sparse defects, $I_{D}$ is proportional to the number of defects within the laser spot. For an average inter-defect distance LD and a laser spot size $L_{L}$, the number of defects probed is approximately ${\left(L_{L}/LD\right)}^{2}$, leading to $I_{D}\propto {\left(L_{L}/LD\right)}^{2}$. Conversely, $I_{G}$ is proportional to the total area illuminated by the laser, i.e., ${{(L}_{L})}^{2}$. Cancado et al. [44] further considered the dependence of the D and G band intensities on the laser excitation energy ${(\lambda }_{L}^{4})$ in the visible range and established the following empirical relationship [44]:

(1) $$L_{D}^{2}[{\mathrm{nm}}^{2}]=(1.8\pm 0.5)\times 10^{-9}\lambda_{L}^{4}{\left(\frac{I_{D}}{I_{G}}\right)}^{-1}$$

where $\lambda_{L}^{4}$ is the laser excitation wavelength. For point-like defects separated by an average distance *Lᴅ* [nm], the defect density $n_{D}$ in ${cm}^{-2}$ can be calculated using the relation [44,50]:

(2) $$n_{D}\left({cm}^{-2}\right)=\left(2.16\times 10^{11}\right)\times \frac{I_{D}}{I_{G}}$$

For a perfect 2D graphene honeycomb lattice, the number of carbon atoms is $n_{C}=\left(3.82\times 10^{15}\right)\;{cm}^{-2}$ [50]. The concentration of Raman-active defects in parts per million (ppm) is obtained from the ratio $\frac{n_{D}}{n_{C}}$ [50]. For sample S2, the measured $\frac{I_{D}}{I_{G}}=1.12$, which leads to $\frac{n_{D}}{n_{C}}=7.8\times 10^{-5}$. Thus, the S2 sample exhibits a Raman-active defect concentration that could be due to the presence of point defects at approximately 78 ppm. Additionally, the $\frac{I_{D}}{I_{G}}$ ratio can also be used to estimate the crystallite size ( $L_{a}$) of *sp*^2^ regions via the Tuinstra–Koenig equation [48]:

(3) $$L_{a}=(2.4\times 10^{-10})\lambda^{4}{\left(\frac{I_{D}}{I_{G}}\right)}^{-1}$$

where  λ is the wavelength of the excitation laser. For the S1 sample, the estimated *Lₐ* is approximately ~17.16 nm, while for the S2 sample, *Lₐ* decreases to ~15.14 nm, reflecting fewer graphene layers due to the post-annealing step. It is known that graphene tends to stack with different layers, which may have resulted in a larger crystallite size observed [8]. Furthermore, the $\frac{I_{2D}}{I_{G}}$ ratio provides information on the number of graphene layers. The 2D band arises from complex electron–phonon interactions and Raman scattering [50]. Its intensity depends on both the number of layers and graphene quality, with the G band serving as a reference. High-quality monolayer graphene typically exhibits $\frac{I_{2D}}{I_{G}}$ ≈ 2 [51], whereas multilayer graphene shows lower values. In our samples, $\frac{I_{2D}}{I_{G}}$ is 0.38 for the S1 sample and 0.67 for the S2 sample, respectively, indicating multilayer structures having structural defects [50].

### 3.4. Magnetic Characterization

**Figure 5:**
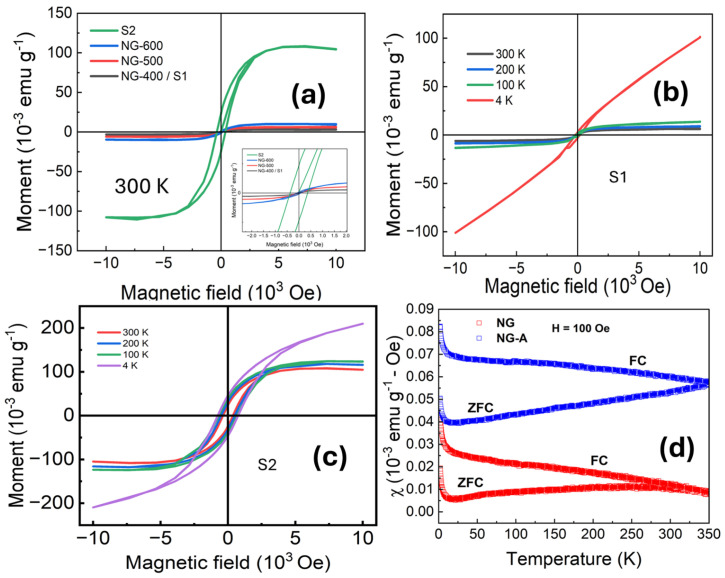
(**a**) Magnetic field curves for S1 and S2 samples measured at 300 K in fields ± 1T. (**b**) M–H curves for the S1 sample at 4, 100, 200, and 300 K in the field ± 1T. A linear trend like paramagnetism is observed at 4 K. (**c**) M–H curves for the S2 sample at the same temperatures as (**b**), showing higher magnetic saturation with evident hysteresis. (**d**) Temperature dependence of magnetic susceptibility for S1 (red) and S2 (blue) samples measured under ZFC and FC at 100 Oe.

Figure 5a presents the magnetic moment versus field curves for NG-400 (S1), NG-500, NG-600 samples, and the post-annealed S2 sample. Notably, the post-annealed S2 sample shows significant improvement in magnetic saturation compared to one-step prepared samples. Quantitatively, the magnetic saturation increases from 0.003 emu g^−1^ to 0.13 emu g^−1^. We observe that NG-600 had lower magnetic saturation as compared to the S2 sample, which was also annealed at 600 °C. As seen in XPS, the overall nitrogen content of the S2 sample decreases with respect to the other NG samples. However, there is an increase in the magnetic saturation of the S2 sample. This suggests that higher magnetic saturation arises not from the higher nitrogen content but rather from the enhanced graphitic-N configuration promoted by the annealing step in Ar atmosphere. As summarized in Table 3, the coercivity also increases noticeably for the S2 sample, from 40 Oe to 750 Oe, respectively. The increase in coercivity indicates that the post-synthesis annealing drives a transition from a superparamagnetic state to a ferromagnetic state. This magnetic transformation is also possibly due to the thermally induced structural defects, as seen by Raman spectroscopy. We observe an increase in remanent magnetization for the S2 sample, indicating enhanced stability of the induced magnetic ordering and stronger magnetic interactions in the system. Interestingly, the NG sample with high content of pyrrolic-N atoms (S1) shows paramagnetic-like behavior, whereas the NG sample with a high content of graphitic-N (S2) shows ferromagnetic behavior. The high saturation moment of the S2 sample is attributed to the graphitic-N contribution.

Magnetization (M–H) curves at various temperatures for S1 and S2 are shown in Figure 5b,c. The S1 sample displays superparamagnetic-like M–H behavior at all measured temperatures except 4 K (Figure 5b), where linearity of the M–H emerges. This linear behavior resembles paramagnetic behavior, as no saturation is achieved even in higher applied fields. As the temperature decreases, a slight reduction in magnetic saturation is observed, accompanied by an increase in coercivity. It is observed that for the S2 sample, the magnetic saturation and coercivity also increase with decreasing temperature (see Figure 5c). The S2 sample exhibits clear hysteresis with higher coercivity, consistent with ferromagnetic-like ordering when measured between 100 K and 300 K. However, at 4 K, the magnetic response became more convoluted, does not reach saturation, and reveals a paramagnetic-like nature. To get more insight, the temperature-dependent magnetic susceptibility (*χ*) measurements in field-cooled (FC) and zero-field-cooled (ZFC) modes under an applied field of 100 Oe were done, which are presented in Figure 5d. S2 sample exhibits consistently higher *χ* values across the entire temperature range (2 K–350 K), with a significant bifurcation between FC and ZFC curves, indicative of magnetic ordering. In contrast, the S1 sample shows lower *χ*, and FC-ZFC splitting occurs at lower temperatures. Both S1 and S2 samples display an upward trend at lower temperatures, suggesting paramagnetic contributions. However, this behavior needs further investigation (in progress).

In our system, magnetic behavior mainly arises from the lattice defects created during the nitrogen-doping process. When fluorinated graphene is thermally treated, fluorine atoms are removed, leaving behind carbon vacancies and unsaturated edge sites. These defect sites act as active centers for nitrogen incorporation, forming different bonding configurations such as pyridinic, pyrrolic, and graphitic nitrogen. Each type of nitrogen contributes in its own way to the overall magnetic behavior, depending on how the electrons are localized and how strongly the defect sites interact with each other. These defects break the symmetry of the graphene lattice and generate localized magnetic moments. This change marks a clear transition from a defect-driven paramagnetic state to a more ordered ferromagnetic phase. The link between nitrogen defect density and magnetic strength is also evident in the Raman ( $\frac{I_{D}}{I_{G}}$) and XPS analyses.

The experimental magnetic measurement results demonstrate that the nitrogen bonding environment strongly influences the magnetic properties of the S1 sample. The S2 sample, with its high graphitic-N content, exhibits strong ferromagnetic-like behavior, while samples rich in pyrrolic-N display primarily paramagnetic-like features. Nitrogen atoms incorporated into five-membered rings, i.e., the pyrrolic-N, adopt an *sp*^3^-like hybridization, giving rise to additional unpaired σ-electrons compared to *sp*^2^-hybridized nitrogen. These localized unpaired electrons generate magnetic moments, but due to the lack of strong interactions between sites, their spins remain randomly oriented, leading to paramagnetic behavior. In contrast, graphitic-N substituting carbon atoms in the basal plane is more directly associated with the emergence of long-range magnetic order. This is because graphitic-N introduces delocalized *π*-electron states that enable spin delocalization and exchange interactions, whereas pyrrolic-N and pyridinic-N remain highly localized and defect-associated, contributing only isolated magnetic moments without cooperative interaction [52,53,54]. We believe that our experimental observations are supported by theoretical predictions as reported in the literature [52,53,54]. In general, the mechanism leading to observed magnetization can be rationalized within the framework of the Ruderman–Kittel–Kasuya–Yosida (RKKY) interaction, where magnetic moments are indirectly coupled through conduction electrons, leading to oscillatory exchange interactions that may be either ferromagnetic or antiferromagnetic depending on interatomic spacing [53,55]. At low nitrogen concentrations, the random distribution of dopant sites prevents net spin alignment, and the overall magnetic response remains weak. However, as the overall nitrogen doping density increases, the average separation between dopants decreases. Beyond a critical threshold, the probability of close N–N spacing rises significantly, promoting cooperative spin alignment and the onset of ferromagnetism in the system. Therefore, to further enhance magnetization in the NG material, one can consider several strategies, including increasing the overall nitrogen density and directing doping toward preferred sites, such as graphitic-N. Higher fluorine content in the starting graphite material may promote more sites for nitrogen incorporation, thereby improving magnetic ordering. In addition, nitrogen incorporation is known to enhance the thermodynamic stability of graphene compared to its undoped counterpart. This stability gain is closely linked to nitrogen content and chemical coordination of pyridinic-N, pyrrolic-N, or graphitic-N, with thermal treatments shifting the balance among these species [56]. Thus, targeted nitrogen doping strategies not only improve magnetic performance but also strengthen the structural robustness of NG materials.

Finally, it is rather clear that due to the complexity of the carbon system, the various configurations of nitrogen site occupations in the carbon matrix caused controversy in establishing a unified point of view at present on the role each specific nitrogen site plays in a particular physical or chemical phenomenon. For example, in the past, XPS experiments attempted to phenomenologically demonstrate if the role of pyridinic-N nitrogen, having lone pair electrons, which, by the delocalization of the *π*-electron from the pyridinic-N is more critical for oxygen reduction reaction as compared to the graphitic-N having nitrogen bonded to three *sp*^2^ carbon atoms [57,58,59]. A similar approach involving XPS measurements supporting magnetic studies seems to be adequate but insufficient to resolve the uncertainty of the contribution of specific nitrogen configurations to the observed magnetism in NG. The role of dominant nitrogen sites or the interaction between various nitrogen sites admixture in enhancing global magnetism of the NG system remains convoluted and debated despite numerous experimental and theoretical efforts. Therefore, the details of the major nitrogen site electron/spin configuration affected by the carbon host lattice, facilitating long-range interaction between sites and the structural defects, need more effort, including X-ray magnetic circular dichroism (experiments are in progress at the SLAC National Accelerator Laboratory) [60].

## 4. Conclusions

This study demonstrates a practical approach for fabricating magnetic nitrogen-doped graphene using commercially available materials. By employing a two-step synthesis process involving ammonia treatment at 400 °C followed by annealing at 600 °C, we achieved a significant transformation in nitrogen-bonding-state densities. We demonstrated that the magnetic behavior of nitrogen-doped graphene is governed by the configuration of nitrogen dopants along with their overall concentration. By combining rapid thermal expansion–exfoliation and chemical transformation at elevated temperatures, we achieved a novel metal-free magnetic carbon material containing a high percentage of graphitic-N sites, promising for room-temperature magnetism. Although the total nitrogen content decreased upon annealing, XPS analysis revealed a substantial increase in graphitic-N, which directly correlated with enhanced magnetic saturation and improved hysteresis. Raman spectroscopy further confirmed defect-induced structural modifications, supported by the presence of D, G, and 2D bands. These findings highlight the critical role of graphitic-N in enabling intrinsic ferromagnetism in carbon-based systems, and offer a promising strategy for engineering sustainable, metal-free magnetic materials suitable for spintronics, memory devices, and other nanoelectronics applications. We believe that we have established a direct correlation between nitrogen dopant configuration and magnetic properties in nitrogen-doped graphene. Our results highlight that graphitic-N plays a pivotal role in stabilizing ferromagnetic-like behavior, offering a new route for designing carbon-based magnetic materials with tunable functionalities. Finally, the proposed approach is a promising and economically viable route for fabricating magnetic NG at high yield, critical for commercialization.

## Figures and Tables

**Table 1:** Elemental Composition (EDS) analysis of S1 and S2 samples.

Element	Line Type	Apparent Concentration		Intensity Correction		K-Ratio		wt. %	
		S1	S2	S1	S2	S1	S2	S1	S2
Carbon	K series	112.02	112.90	0.86	0.89	1.12022	1.12897	85.38	89.56
Nitrogen	K series	4.59	2.77	0.26	0.25	0.00817	0.00493	11.59	7.86
Oxygen	K series	0.72	0.65	0.19	0.19	0.00241	0.00219	2.46	2.36
Fluorine	K series	0.44	0.31	0.51	0.52	0.00085	0.00060	0.56	0.22

**Table 2:** Elemental and nitrogen bonding composition in S1 and S2 samples.

**Sample**	**C at.%**	**O at.%**	**F at.%**	**N at.%**		
NG-400 (S1)	58.3	27.2	2.6	11.9		
				%N_Pyrrolic_	%N_Pyridinic_	%N_Graphitic_
				49.3	27.2	23.5
NG-500	65.1	20.2	2.2	12.5		
				%N_Pyrrolic_	%N_Pyridinic_	%N_Graphitic_
				37.5	36.3	26.2
NG-600	75.2	10.6	1.5	12.7		
				%N_Pyrrolic_	%N_Pyridinic_	%N_Graphitic_
				30.5	41.2	28.3
S2	89.7	4.6	0.2	5.5		
				%N_Pyrrolic_	%N_Pyridinic_	%N_Graphitic_
				15.6	23.8	60.6

**Table 3:** Magnetic properties of various NG samples obtained from VSM measurements.

Sample	N (at.%)	M_s_ (10^−3^ emug^−1^)	H_c_ (10^3^ Oe)	M_r_ (10^−3^ emug^−1^)
NG-400 (S1)	11.9	3.06	0.04	0.29
NG-500	12.5	5.09	0.15	0.79
NG-600	12.7	11.80	0.15	0.79
S2	5.5	129.81	0.75	23.71

## Data Availability

Data is contained within the article and Supplementary Materials.

## Supplementary Materials

The following supporting information can be downloaded at: https://www.mdpi.com/article/10.3390/nano15221694/s1, Figure S1: M-H curves measured for different NG samples with synthesis parameters; Table S1: Parameters used for the optimization of NG materials during the two-step synthesis; Figure S2: (a) Survey spectrum of NG-500 (b) High resolution of N1s spectra of NG-500 (c) Survey spectrum of NG-600 (d) High resolution of N1s spectra for NG-600; Figure S3: The deconvoluted Raman spectra for (a) S1, (b) S2.
