# Supplementary material for: Magnetic Properties of Nitrogen-Doped Graphene Induced by Dopant Configurations
Source: Nanomaterials (Basel). 2025 Nov 9;15(22):1694. doi: 10.3390/nano15221694 (PMC12655189; doi:10.3390/nano15221694)
Supplement: Supplementary file 1 [file nanomaterials-15-01694-s001.zip › nanomaterials-3936704-supplementary.pdf]

<u>**Supplementary Materials**</u>

Magnetic Properties in Nitrogen-Doped Graphene Governed by Dopant Configurations

Madhuparna Chakraborty[1*], Gregory Jenson[2], David C. Ingram[2], Eric Stinaff[2], and Wojciech M. Jadwisienczak[1]
[1]School of Electrical Engineering and Computer Science, Ohio University, Athens, OH 45701, USA
[3]Department of Physics and Astronomy, Ohio University, Athens, OH 45701, USA
[*]mc559517@ohio.edu

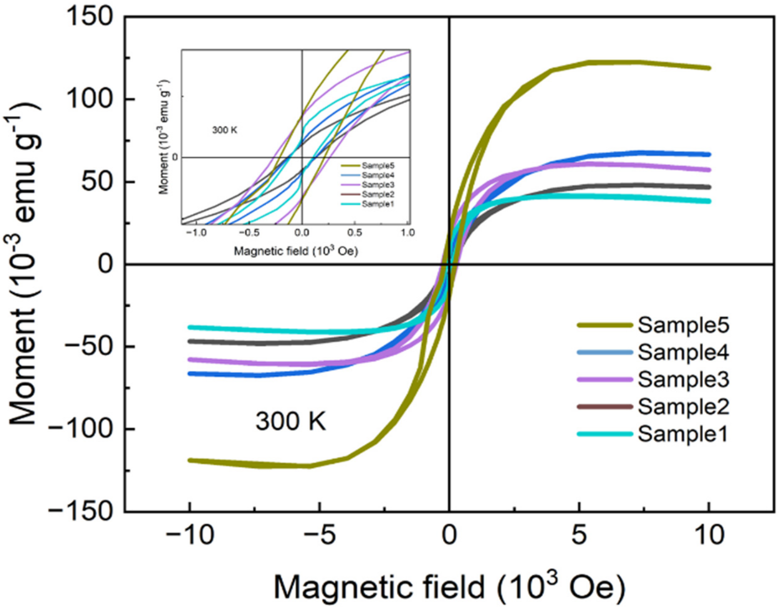

Figure S1. M-H curves measured for different NG samples with synthesis parameters shown in Table S1.

Figure S1 shows room temperature magnetization M-H curves of annealed NG-500 and NG-600 samples, along with S2 samples, which was discussed in the main text because of the enhanced magnetization. The annealing of NG-500 and NG-600 resulted in deterioration of magnetic saturation compared to the S2 sample.

Table S1. Parameters used for the optimization of NG materials during the two-step synthesis.

**Sample**	**Input parameters**		**Output parameters**		
	**Synthesis temperature (°C)**	**Annealing temperature (°C)**	**Saturation magnetizatio n (emu g^{-1})**	**Coercivity (Oe)**	**Remanance (emu g^{-1})**
Sample 0 / (S1)	400	-	0.003	40	0.0002
Sample 1	500	700	0.039	134.48	0.0060
Sample 2	500	650	0.048	110.44	0.0050
Sample 3	600	800	0.057	269.75	0.0080
Sample 4	600	700	0.068	125.32	0.0060
Sample 5 / (S2)	400	600	0.130	235.87	0.0100

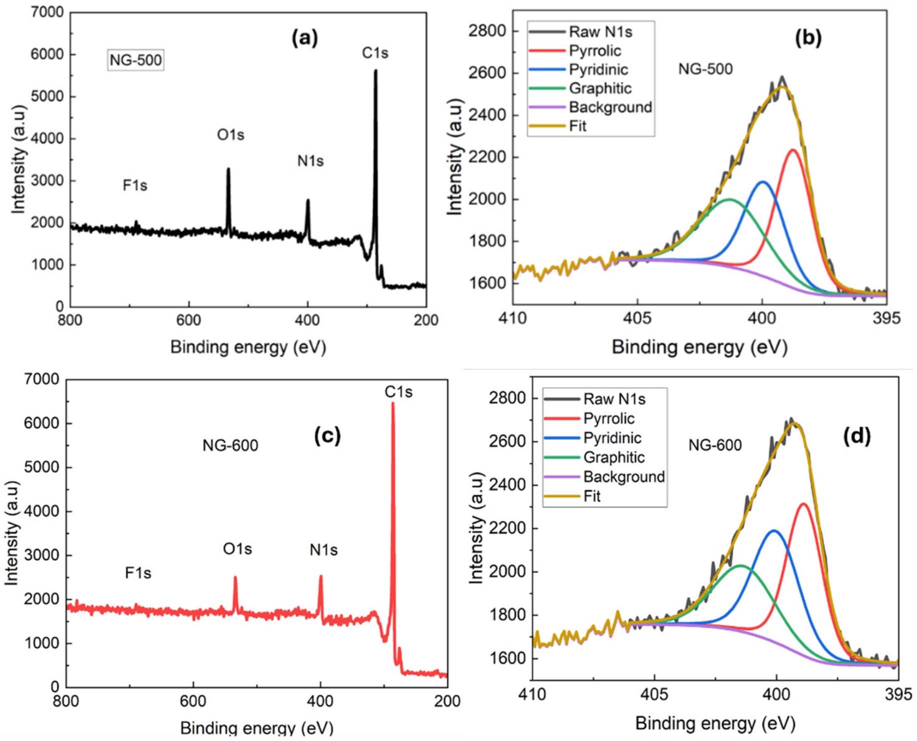

Figure S2. (a) Survey spectrum of NG-500 (b) High resolution of N1s spectra of NG-500 (c) Survey spectrum of NG-600 (d) High resolution of N1s spectra for NG-600

NG-500 and NG-600 refer to samples that did not undergo annealing. The survey spectra in Figure S2(a) and (c) show a decrease in nitrogen, oxygen, and fluorine content with increasing reaction temperature from 500 °C to 600 °C. The N 1s spectra in Figure S2(b) and (d) further indicate that pyrrolic-N is the dominant configuration in both samples.

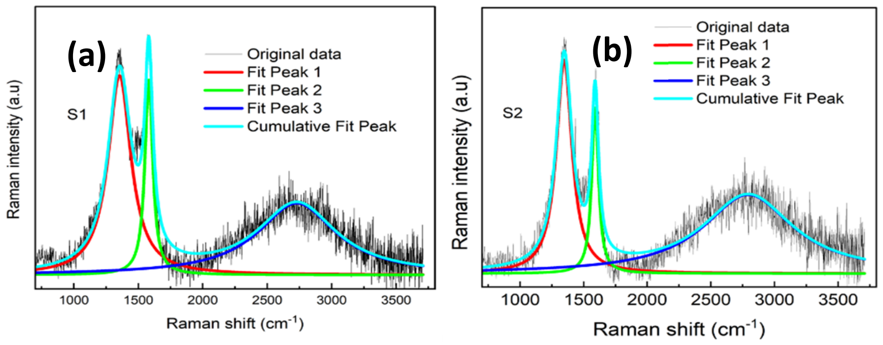

Figure S3. The deconvoluted Raman spectra for (a) S1, (b) S2

To determine the $\frac{I_D}{I_G}$ ratio, the Raman spectra of S1 and S2 were fitted using three Lorentzian peaks centered at ~1352 cm^{-1} (D band), ~1589 cm^{-1} (G band), and ~2749 cm^{-1} (2D band). The corresponding fitted spectra are shown in Figure S3(a) and (b). The quality of the fits was high, with adjusted R^2 values of ~0.97 or greater.
